# The new age of the phage

**DOI:** 10.1042/EBC20240037

**Published:** 2024-12-17

**Authors:** Joanne M. Santini

**Affiliations:** Structural & Molecular Biology, Division of Biosciences, UCL, London, U.K.

**Keywords:** antibiotics, bacteriophages, pathogens, therapeutics

## Abstract

The discovery of viruses that can devour bacteria, bacteriophages (phages), was in 1915. Phages are ubiquitous, outnumbering the organisms they devour, and genomically, morphologically, and ecologically diverse. They were critical in our development of molecular biology and biotechnology tools and have been used as therapeutics for over 100 years, primarily in Eastern Europe with thousands of patients from all over the world treated in Georgia. The rise of antimicrobial resistance and the lack of new antimicrobials, has brought them back into the spotlight dawning the New Age of the Phage. This special issue will provide further insight to phage diversity across ecosystems, including humans, animals, and plants, i.e. the basis of a One Health approach, and the requirements for turning phages into viable medicines for the many and not just for the few.

Bacteriophages (phages) are viruses that infect bacteria. In general, phages can be virulent, killing the cells, or temperate (lysogenic), integrating into bacterial chromosomes as prophages and upon exiting can enter a lytic phase killing the bacteria. Phages were discovered by William Twort in 1915 and Felix d’Herelle demonstrated their ability to kill bacteria in 1917. This discovery led to their therapeutic use (phage therapy) across Europe until the disovery of antibiotics. From 1923 the medicinal use of phages continued in Eastern Europe with Georgi Eliava at his institute, now known as the George Eliava Institute of Bacteriophages, Microbiology and Virology [1]. The Institute celebrated its 100th anniversary last year and has treated thousands of patients from across the world. Although phage therapy’s inception was over 100 years ago, a detailed mechanistic understanding of their use in therapy is however in its infancy. What is clear from more than a century of research is that they appear safe and can work to treat bacterial infections. Fundamental phage research continued in the West where phages proved critical to the development of molecular biology and biotechnology tools, examples including genomic library construction, sequencing, phage display, and more recently in gene editing and in diagnostics.

Phages are ubiquitous, found in any environment you find bacteria. They are hugely diverse, varying in size (from 3.5 to over 700 kbp), genomically, morphologically, in the hosts they infect, their lifestyle and in their ecological niche. Identifying phages is not always clear cut especially from metagenomics data. Other mobile elements exist that display similarities between plasmids and prophages. These mobile genetic elements, known as phage-plasmids is the topic of the review by Sayid et al. [2]. Phage-plasmids are known to carry genes involved in lysogeny and lysis as well as genes involved in plasmid replication, partitioning allowing for organised segregation tied to cell division, and some conjugation functions allowing them to be mobilised from one bacterium to another. Transfer of genes can occur between phage-plasmids and plasmids, and to a lesser extent with phages. Like plasmids and phages, phage-plamids can also mediate the transfer of genes that increase pathogenicity (e.g. virulence genes) and antimicrobial resistance of organisms as well as bacterial resistance against other mobile genetic elements. It is currently unclear what the impact of bacterial resistance from molecules such as chromosomes, prophages, phage-plasmids or plasmids have on phage therapy.

Pre-2019 our view of phage diversity was mostly limited to phages that were cultured. The application of genome-resolved metagenomics techniques to phages resulted in not only the discovery of the first megaphages (with genomes greater than 500 kb) but also to the discovery of many more jumbo phages (>200 kb) especially in the gastrointestinal tract of humans and a variety of animals [3,4]. This new approach to phage discovery has led to more than doubling of phage genomes, especially in environments which were previously underexplored such as gut microbiomes. This increased exponentially our understanding of phage genomics and diversity. Many of these phages remain unisolated for a number of reasons. The review by Xiao et al. covers what we know about phages in the gastrointestinal (GI) tract highlighting the clear paucity of phages isolated against obligate anaerobes which dominate the human GI tract [5]. They suggest different approaches to isolate phages, highlighting the bias in methods towards phages that infect aerobes with either no or few phages against common obligate anaerobes (e.g. *Segatella copri*). There have however been a large number of phages identified and even isolated that fall into the new family of abundant *Crassvirales*, some of which target the obligate anaerobe *Bacteroides* as their host. Xiao et al. also discuss the therapeutic uses of gut phages, for example, through targeting organisms implicated in inflammatory bowel disease and in drug delivery to treat colorectal cancer (CRC), although their potential use against bacteria implicated in CRC has not yet been explored [5].

Beyond the GI tract of humans there are a multitude of genetically distinct phages for individual bacterial strains and this variation means that they can be employed as antimicrobial agents for a wide variety of pathogens. Understanding how they can be employed in the One Health (across ecosystems including in humans, animals, and plants) context is going to expand their use beyond humans. Their specificity means they can be employed not just therapeutically but prophylactically as antimicrobials in the One Health context. The review by Adriaenssens et al. summarises their use across One Health showcasing some examples of use within the food safety sector and human health particularly [6]. The majority (207 out of 211) of phages that have been used therapeutically are from a single class of phages, the *Caudoviricetes*, compared to the >28,000 sequenced isolates and the tens of thousands that have not yet even been isolated [6]. Given the genomic, physiological, and ecological diversity of pathogens and the renewed interest in phages as antimicrobials, it is expected that new approaches to phage isolation will be needed to tackle One Health problems.

The rise of antimicrobial resistance and new regulations curbing the overuse of antibiotics in the environment and animal husbandry has resulted in the increased interest in the use of phages. Schooley delivers a comprehensive review of the current state of phage therapy, where the reproducibility of testing phage susceptibility is a major issue, resulting in discordant results from the same laboratories [7]. Standardisation is clearly going to be critical. Other factors that require consideration are dosage, formulation, stability, and delivery, which will probably vary depending on the phage, bacterium, and the individual host. All these factors need to be considered prior to their use in therapy and will depend on the site of their deployment. Ando et al. highlight in their review the importance of the host immune response in clearing phages and the production of phage-specific antibodies, particularly if phages are deployed intravenously [8]. All these factors make it difficult to understand the pharmacokinetics and pharmacodynamics. They highlight avenues of promoting the pharmacokinetics by, for example, glycosylation of the phages. Alternative ways of protecting the phages could also include methods deployed for bacterial therapeutics [9]. What is clear from the evidence in the literature is that phages are considered generally safe. Other important considerations that Schooley’s [7] review highlights as critical to the success of phage therapy include the need for longitudinal microbiological assessment to determine, for example, if antiphage resistance results in therapy failure. Given that antiphage resistance is broadly found not only on bacterial chromosomes but also mobile elements including plasmids, prophages, and even phage-plasmids, evidence to whether they play a role in therapy failure is going to be key. Engineering phages to overcome bacterial resistance and/or the design of synthetic phages using computational approaches is an emerging transformative technology for phage therapeutics.

Another approach to the use of phages as antimicrobials is to use phage enzymes. Endolysins, which degrade the peptidoglycan layer of bacteria, have been the most extensively studied and their therapeutic use is now well established. Briers et al. highlight that key to the development of endolysins as therapeutics is their production [10]. Different expression platforms have been developed, with the chosen method depending on final use and with several considerations taken into account, such as the presence of endotoxins, solubility, toxicity, and cost. Endolysins are currently not used on all pathogens, with capsulated isolates being particularly challenging. Here, a different suite of phage enzymes, namely depolymerases, can be deployed to degrade the bacterial capsules with their potential therapeutic use in combination with other antimicrobials such as antibiotics, endolysins or even phages. Santini et al. review the diversity of depolymerases against the WHO priority group 1 pathogen *Klebsiella pneumoniae* that is not only genomically diverse but has >80 known capsule (K) types [11]. The mechanisms of depolymerase action against the different K types is poorly understood and will be key to determining not only how best to use these enzymes as therapeutics but whether there is a depolymerase that can work on all (one that decapsulates them all) or many K types or indeed whether depolymerases can be engineered to be broader spectrum. At the moment the only therapeutic route is to use depolymerase cocktails as is done for phages, and possibly in combination with antibiotics, however given the diversity of *K. pneumoniae* K types even in a single hospital this does not seem a viable route to successful therapy or commercialisation.

The threat of increasing deaths and prolonged illness caused by AMR means that alternative therapies are essential. As reviewed by Edwards et al. health research prioritisation is determined by a metric known as the disability-adjusted life year (DALY) which measures the global impact of a disease or condition [12]. Edwards and colleagues explain that method standardisation will be essential in forming a universal regulatory fraemework that integrates phage therapy into mainstream medical practice. Critical to this will be longitudinal data providing further evidence for safety and most importantly evidence about efficacy. The progression of phage therapy beyond a case by case basis for use in compassionate cases to clinical trials is going to require clear regulatory guidelines and a standardised framework for testing phage efficacy.

Over a century of research on phages has highlighted their importance, not only in shaping microbial communities in different ecological niches, but also in both biotechnology and medicine. Their ability to selectively target bacteria makes them an extraordinary tool for both therapeutics and microbiome engineering. Critical to their therapeutic use is the requirement for clear regulatory guidelines, which at the moment vary worldwide with some countries having no guidelines. In the UK, we look to the future of phage therapy by highlighting the recent UK government Commons Science Innovation and Technology Committee report [13]. This report published in March 2024 eloquently summarises the barriers and other considerations required for the successful implementation of phage therapy [13]. What is clear from the collection of reviews in this special issue is that a unified approach to tackle AMR is needed across the One Health sector and that phages (and their enzymes) have an essential role to play in achieving this.

## Open Access

Open access for this article was enabled by the participation of University College London in an all-inclusive *Read & Publish* agreement with Portland Press and the Biochemical Society under a transformative agreement with JISC.

## Abbreviations

AMR: antimicrobial resistance
CRC: colorectal cancer
DALY: disability-adjusted life year
GI: gastrointestinal

## References

[1] The Eliava Institute. (2024) https://eliava-institute.org/Accessed 19 November 2024.

[2] Sayid R., van den Hurk A.W.M, Rothschild-Rodriguez D., Herrema H., de Jonge P.A., Norbega F.L. et al. (2024) Essays Biochem. 68 5 583–592, 10.1042/EBC20240014 39611587 PMC11652158

[3] Devoto A.E., Santini J.M., Olm M.R., Anantharaman K., Munk P., Tung J. et al. (2019) Megaphages infect Prevotella and variants are widespread in gut microbiomes. Nat. Microbiol. 4, 693–700 10.1038/s41564-018-0338-9 30692672 PMC6784885

[4] Al-Shayeb B., Sachdeva R., Chen L.-X., Ward F., Munk P., Devoto A. et al. (2020) Clades of huge phages from across Earth’s ecosystems. Nature 578, 425–431 10.1038/s41586-020-2007-4 32051592 PMC7162821

[5] Liu C., Xing B., Li Z., Li J. and Xiao M. (2024) A roadmap of isolating and investigating bacteriophage infecting human gut anaerobes. Essays Biochem. 68 5 593–605 10.1042/EBC20240116 39611592 PMC11652169

[6] Pye H.V., Krishnamurthi R., Cook R. and Adriaenssens E.M. (2024) Evolution and diversity of phages. Essays Biochem. 68, 5, 607–619 10.1042/EBC20240012 39475220 PMC12055037

[7] Schooley R.T. (2024) Translational research priorities for bacteriophage therapeutics. Essays Biochem. 68, 5,621–631 10.1042/EBC20240020 39417290 PMC11652170

[8] Washizaki A., Sakiyama A. and Ando H. (2024) Phage-specific antibodies: are they a hurdle for the success of phage therapy? Essays Biochem. 68, 5,633–644 10.1042/EBC20240024 39254211 PMC11652166

[9] Cao Z. and Liu J. (2024) Surface nanocoating of bacteria as a versatile platform to develop living therapeutics. Nat. Protoc. 19, 3162–3190 10.1038/s41596-024-01019-6 39044001

[10] Cremelie E., Vázquez R. and Briers Y. (2024) A comparative guide to expression systems for phage lysin production. Essays Biochem. 68, 5,645–659 10.1042/EBC20240019 39290148 PMC11652153

[11] Cheetham M.J., Huo Y., Stroyakovski M., Cheng L., Wan D., Dell A. and Santini J.M. (2024) Essays Biochem. 68 5 661–677 10.1042/EBC20240015 39668555 PMC11652154

[12] Edwards S.J.L., Tao Y., Elias R. and Schooley R. (2024) Considerations for prioritising clinical research using bacteriophage. Essays Biochem. 68, 5,679–686 10.1042/EBC20240013 39475266 PMC11652150

[13] Department of Health and Social Care (2024) Government's response to the Science, Innovation and Technology Committee's report ‘The antimicrobial potential of bacteriophages’ Available at: The antimicrobial potential of bacteriophages report: government response Accessed 19 November 2024.

